# Secondary Pneumothorax Induced by a Bronchopleural Fistula in a Patient With COVID-19 Pneumonia

**DOI:** 10.7759/cureus.26627

**Published:** 2022-07-07

**Authors:** Connor Smith, Olivia Mobarakai, Amber Bux, Ralph Kamel, Neville Mobarakai

**Affiliations:** 1 Internal Medicine, Touro College of Osteopathic Medicine, New York, USA; 2 Internal Medicine, Staten Island University Hospital, Northwell Health, New York, USA; 3 Infectious Disease, Staten Island University Hospital, Northwell Health, New York, USA

**Keywords:** pneumonia, empyema, bronchopleural fistula, pneumothorax, covid-19

## Abstract

COVID-19 is a multi-system disease caused by severe acute respiratory syndrome-coronavirus 2 (SARS-CoV-2). One of the main highlights of the disease is the development of pneumonia complicated by adult respiratory distress syndrome. While spontaneous pneumothorax has been reported in some patients with COVID-19, bronchopleural fistula has seldom been reported as the primary cause in these cases. We describe the rare case of a young patient who developed a pneumothorax complicating COVID-19 and was found to have a bronchopleural fistula and empyema secondary to *Staphylococcus aureus* superinfection.

## Introduction

Novel severe acute respiratory syndrome-coronavirus 2, also known as coronavirus disease 19 (COVID-19), is a rapidly progressive illness with various fatal respiratory complications. Spontaneous pneumothorax is defined as an accumulation of gas in the pleural space. A bronchopleural fistula is defined as a sinus tract between the bronchi and pleura, resulting in an empyema, or pus collection in the pleural cavity, and is associated with very high mortality. While cases of spontaneous pneumothorax in COVID-19 patients have been reported, cases of bronchopleural fistula are rare. In this report, we present one of the rare literature-described cases of bronchopleural fistula and empyema formation secondary to *Staphylococcus aureus* in a patient with a recent history of COVID-19 infection.

## Case presentation

**Figure 1:**
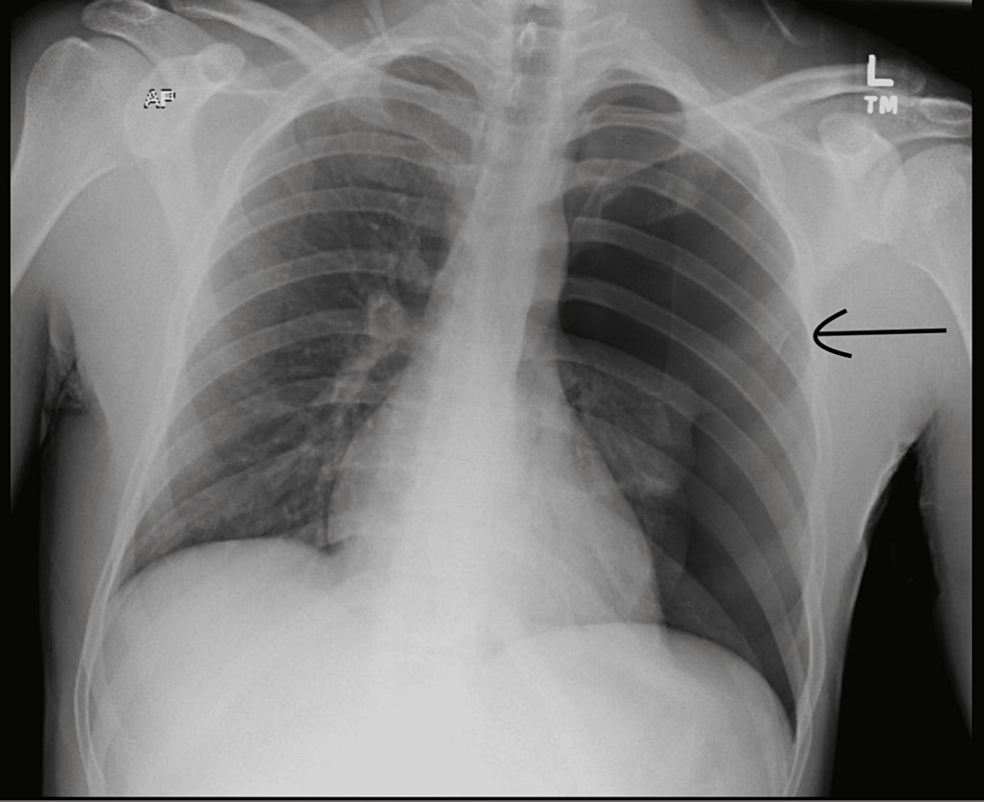
Large left pneumothorax with complete left lung collapse

A 26-year-old Caucasian male with no past medical history presented to our emergency department complaining of left-sided chest pain for the past two days. He had been diagnosed with COVID-19 by PCR 4 weeks prior. Before the onset of chest pain, his symptoms had been limited to anosmia and ageusia. He denied shortness of breath. His vitals were temperature (F) 97.3, heart rate (HR) 67, blood pressure (BP) 124/72, respiratory rate (RR) 18, and saturation of 98% on room air. Chest X-ray demonstrated a large left pneumothorax with a complete collapse of the left lung (Figure 1). A left-sided pigtail catheter was placed without complication and put to suction. Physical examination revealed left chest wall tenderness but was otherwise unremarkable. The patient was started on acetaminophen 650 mg PO q6h PRN, ketorolac 30 mg IV push q8h PRN, and oxycodone IR 5 mg PO q6h PRN for pain control. He was admitted to the thoracic surgical unit for observation.

Several days following chest tube placement, chest X-rays demonstrated resolution of the pneumothorax and complete expansion of the lung. The patient was placed on 2 L supplemental oxygen via nasal cannula. He was encouraged to ambulate and cooperated with the use of the incentive spirometer. Of note, his COVID PCR tests on Day 1 and Day 6 were both negative. On Day 6 of his hospitalization, a decision was made to switch the patient’s chest tube from suction to a water seal, given his marked clinical improvement and a recent decline in his chest tube output to minimal levels. A CXR the following morning showed recurrence of the large left pneumothorax with an air gap of 5.5 cm. Despite the improvement, the surgical team decided to proceed with operative intervention.

On Day 8, the patient underwent video-assisted thoracoscopy, left upper lobe wedge resection, and mechanical pleurodesis. The patient was noted to have a left-sided bronchopleural fistula during the procedure. A 28 French left apical chest tube was inserted. The patient received 2 g of IV Ancef. The patient tolerated the procedure well and returned to the cardiothoracic unit for postoperative supportive care and monitoring. The following day, the patient’s temperature rose to 102.6ºF, and CXR showed a trace pneumothorax. The white blood cell count was 10.78 k/µL. Pleural fluid culture grew methicillin-sensitive *S. aureus* and was considered by the infectious disease consultant to constitute an empyema. The patient was started on Ancef 2 g IV q8h for 10 days, to then be switched to dicloxacillin 500 mg PO q6h and probenecid PO 500 mg q12h. On Day 14, CXR showed complete resolution of the pneumothorax, and the patient was discharged home on the aforementioned antibiotic regimen.

## Discussion

Spontaneous pneumothorax is most frequently observed in tall, thin male patients aged between 10 and 30 years. The etiology is thought to be the formation of apical pleural blebs that develop due to increased negative pressure and mechanical alveolar stretch at the apex of the lungs [1]. More common in the elderly population, secondary spontaneous pneumothorax is caused by underlying lung diseases such as chronic obstructive pulmonary disease (COPD), tuberculosis, and rarely pneumonia [2]. Notably, and in contrast to our case, pneumothorax is rarely an initial manifestation of these diseases and usually presents late in the disease course [3].

Several case series have described COVID-19 presentations with pneumothorax, with one review of over 3,000 COVID-positive patients showing a pneumothorax prevalence of 0.66% [4]. A separate review of 25 case reports found that most cases were in patients of advanced age, with multiple comorbidities, and who developed pneumothorax while critically ill, almost all eventually requiring mechanical ventilation [5]. These demographics stand in sharp contrast to our patient’s.

Only one other case report describes a patient resembling ours: a young man with mild symptoms of COVID-19 who did not require hospitalization until the pneumothorax had already developed. Unlike our patient, that case was resolved within 48 hours of chest tube placement and successfully transitioned from suction to water seal after 24 hours [6]. As described, our patient experienced a recurrence of the pneumothorax after switching to a water seal, and after seven days of persistent positive chest X-rays, he required mechanical pleurodesis.

The cause of this patient’s persistent pneumothorax was revealed during the thoracoscopy to be a bronchopleural fistula. As mentioned above, a bronchopleural fistula is an aberrant connection between a bronchus and the pleural space, allowing the buildup of air in the pleural cavity, leading to pneumothorax. The etiology of the bronchopleural fistula is most related to lung resection, malignancy, and other mechanical insults to the lung parenchyma. Rarely is it due to inflammatory processes of the lung.

Only one other case report has described a patient with bronchopleural fistula and COVID-19 [7]. That patient, a 49-year-old male with no significant past medical history, presented with fever, shortness of breath, and significant hypoxia. He was hospitalized for 21 days, had seven days of mechanical ventilation, and developed septic shock secondary necrotizing infection with mucormycosis. That patient expired despite aggressive antimicrobial and vasopressor therapy as well as pleurodesis for the fistula. While our patient had a significantly more benign presentation and hospital course, he also developed a fistula with empyema, which tested positive for methicillin-sensitive *S. aureus*.

Our report further supports a small pool of evidence linking COVID-19 pneumonia, spontaneous pneumothorax, and bronchopleural fistula. It describes an unusually young and healthy COVID patient developing spontaneous pneumothorax and one of only two reported cases of bronchopleural fistula. Because this patient’s COVID-19 diagnosis was four weeks old at the time of presentation, it seems likely that he had subacute COVID pneumonia with overlying *S. aureus* empyema, creating an inflammatory environment that allowed the fistula to develop and progress to a pneumothorax. Fortunately, his mechanical pleurodesis was successful, and his pleural fluid grew a pathogen susceptible to common antibiotics. It is hard to refute that his youth and lack of comorbidities played an essential role in his recovery. This report should remind clinicians that COVID-19 pneumonia can lead to unexpected and severe pulmonary complications and those outcomes are heavily dependent on the qualities of both the pathogens and the patient.

## Conclusions

The development of pneumothorax in COVID-19 is well documented in the literature; however, bronchopleural fistula has seldom been reported as the etiology. Our case report highlights the importance of considering this etiology in a patient with COVID-19 presenting with a recurrent pneumothorax. Early identification is crucial given the potential for life-threatening complications such as the development of empyema.
